# Evaluating JPEG2000 compression effects on prediction accuracy and storage efficiency in lung cancer whole-slide images: a deep learning–based analysis

**DOI:** 10.1186/s13000-026-01830-7

**Published:** 2026-08-11

**Authors:** Hyeseong Ji, Moses Yook, Chan Kwon Jung, In Young Choi

**Affiliations:** 1 Department of Precision Medicine and Big Data, College of Medicine, The Catholic University of Korea, 222 Banpo-daero, Seocho-gu, 06591 Seoul, Republic of Korea; 2 Department of Medical Informatics, College of Medicine, The Catholic University of Korea, 222 Banpo-daero, Seocho-gu, Seoul, 06591 Republic of Korea; 3 Department of Hospital Pathology, College of Medicine, The Catholic University of Korea, 222 Banpo-daero, Seocho-gu, Seoul, 06591 Republic of Korea

**Keywords:** Digital pathology, Whole slide image, JPEG2000 compression, Lung adenocarcinoma, Deep learning, Image segmentation, Storage efficiency, Vision Transformer, Convolutional neural network

## Abstract

**Background:**

Whole-slide images (WSIs) in digital pathology are generated at gigapixel resolution, leading to substantial storage and data management demands. Lossy image compression is commonly used to mitigate these challenges. However, its impact on deep learning (DL)–based image analysis, particularly for detailed segmentation tasks, remains insufficiently characterized. DL models may be sensitive to subtle compression artifacts, underscoring the need for systematic evaluation.

**Methods:**

We evaluated JPEG2000 compression effects on tile-level tumor segmentation using lung adenocarcinoma WSIs. Eighty-six WSIs annotated by four board-certified pathologists were subdivided into nonoverlapping 512 × 512 tiles at 20× magnification. To assess architectural robustness, we trained CNN-based models (U-Net with MobileNetV3, ResNet-18, ResNet-50 encoders) and a Transformer-based model (SegFormer) exclusively on lossless PNG images. These models were evaluated on test tiles compressed using JPEG2000 at rates ranging from 1 (lossless) to 80. Segmentation performance was assessed using the Dice coefficient. Image quality was quantified using Peak Signal-to-Noise Ratio (PSNR), Structural Similarity Index Measure (SSIM), and the no-reference Perception-based Image Quality Evaluator (PIQE). Storage efficiency was evaluated relative to uncompressed TIFF images.

**Results:**

Segmentation performance remained stable at mild compression levels, with a marginal decrease of approximately 1.7% observed in the most robust model (MobileNetV3) up to JPEG2000 compression rates of 10 compared with the lossless baseline. In this range, image quality metrics remained high (PSNR ≥ 33 dB, SSIM ≥ 0.97), while storage requirements were reduced by approximately 90%. At higher compression rates (≥ 15), segmentation performance declined progressively, accompanied by marked degradation in image quality and increased perceptual artifacts. Comparative analysis across model architectures revealed that convolutional neural network (CNN)–based models exhibited greater robustness to compression artifacts than Transformer-based models, which demonstrated an earlier and steeper performance decline. Analysis of model confidence maps further showed that boundary uncertainty increased with compression severity, even before substantial Dice score degradation became apparent.

**Conclusions:**

JPEG2000 compression at mild rates preserves DL-based segmentation performance while substantially reducing storage requirements in digital pathology workflows. These findings provide quantitative evidence on compression-performance trade-offs for tile-level image analysis under controlled experimental conditions and highlight architecture-dependent sensitivity to compression artifacts.

## Background

In digital pathology, whole-slide images (WSIs) are routinely generated at gigapixel resolution, resulting in individual slide files ranging from several hundred megabytes to multiple gigabytes. At 40× magnification, a typical WSI has a resolution of approximately 0.25 μm per pixel and 24-bit color depth, producing up to 48 MB of image data per 1 mm² of tissue without compression [1]. Given that a typical tissue section covers hundreds of square millimeters, the resulting uncompressed file size can easily reach multiple gigabytes. Such ultrahigh resolution imposes significant storage and transmission demands, especially in clinical settings, where large volumes of slides are scanned daily. This challenge is further amplified in multi-institutional environments [2].

To address these limitations, most WSIs are stored in a multiresolution pyramid architecture, in which each resolution level is divided into fixed-size tiles and saved using lossy compression formats such as Joint Photographic Experts Group (JPEG) or JPEG2000 [2]. This structure supports selective region retrieval at different magnifications, thereby improving navigation and reducing storage and transmission costs. However, lossy compression degrades fine structural details, which can affect both the diagnostic accuracy and performance of computational pathology models [3, 4].

In radiology, lossy compression is conditionally accepted when the image quality meets the clinical thresholds supported by validation studies [5–8]. However, compression standards established for radiology cannot be directly transferred to digital pathology. Unlike radiological scans, WSIs are characterized by gigapixel resolution, full-color information, and a critical reliance on fine microscopic textures (e.g., chromatin patterns). Therefore, verifying compression tolerance specifically for WSI is essential. Moreover, pathology workflows generate up to 1,000 times more image data than radiology workflows, demanding significantly greater computational resources, scalable storage infrastructure, and high-speed networking systems [9]. As the adoption of digital pathology grows, the need to balance image fidelity and storage efficiency in AI-based workflows is becoming increasingly important.

Several clinical studies have examined the diagnostic tolerance for lossy compression from the perspective of pathologists. Kalinski et al. [10] reported no significant reduction in diagnostic accuracy in gastric biopsies with JPEG2000 compression of up to 20:1. Similarly, Krupinski et al. [3] reached the same conclusion using breast biopsy slides, observing stable performance even at 32:1 compression. Furthermore, Marcelo et al. [11] demonstrated in a randomized clinical trial that JPEG compression, which reduced file sizes by approximately 90%, did not significantly affect diagnostic accuracy or confidence levels.

These results suggest that moderate compression is well-tolerated in human analysis. However, Deep Learning (DL) models may respond differently. While pathologists use contextual cues to filter out artifacts, DL algorithms often rely on pixel-level statistics and high-frequency edge information, making them potentially more susceptible to subtle artifacts that remain imperceptible to the human eye.

Similar patterns have emerged in DL applications, though with task-specific variances. Chen et al. [4] evaluated the effect of compression on three specific use cases: nuclei segmentation, lymph node metastasis segmentation, and lymphocyte detection. They showed that performance remained stable with JPEG and JPEG2000 compression ratios up to 10:1 (corresponding to PSNR values of 30–35 dB). Zanjani et al. [12] further confirmed, using the CAMELYON16 dataset (breast cancer lymph node metastasis), that DL performance remained stable up to a JPEG2000 compression ratio of 24:1, with notable degradation observed beyond 32:1.

However, previous studies have primarily focused on breast pathology or specific detection tasks. Since histopathological characteristics—such as the complex glandular architecture of lung adenocarcinoma—vary significantly by disease, compression tolerance established in other tissues may not be directly transferable. There remains a limited understanding of how lossy compression affects detailed segmentation tasks in lung adenocarcinoma.

To address this knowledge gap, this study evaluated the effects of varying JPEG2000 compression rates on the tile-level segmentation performance of DL models using histopathological images of lung adenocarcinoma. The analysis was performed on 512 × 512 image tiles extracted at a diagnostic resolution (20×) that is commonly used in AI training pipelines. Although this approach does not account for full-slide contextual reasoning, it provides practical insights into the impact of compression on tile-level image-analysis performance. This study used lung adenocarcinoma slides and quantified the effects of JPEG2000 compression on tile-level predictions to inform AI-based storage strategies rather than for direct diagnostic use.

## Materials and methods

### Dataset and annotation

This study was approved by the Institutional Review Board of Seoul St. Mary’s Hospital, The Catholic University of Korea. We obtained a dataset of lung adenocarcinoma WSIs from the pathology archive of the hospital. A total of 86 hematoxylin and eosin (H&E)-stained WSIs were scanned at 40× optical magnification using a NanoZoomer scanner (Model C13220, Hamamatsu, Shizuoka, Japan) and stored in the NanoZoomer digital pathology image format. All slides had a consistent spatial resolution of 0.2297 μm/pixel, as confirmed using OpenSlide metadata software [2], thereby enhancing the reproducibility of the experiments.

Tumor regions were annotated by four board-certified pathologists. To ensure consistency, each WSI was cross-reviewed by at least two pathologists, and the finalized consensus annotations were used as the reference standard.

### Tile extraction and preprocessing

Level 0 (40×) resolution was excluded as 20× magnification is generally considered sufficient for standard histopathological interpretation, including routine analysis of H&E and IHC slides [1]. Therefore, we focused on Level 1 resolution (approximately 20× magnification), which is widely adopted in clinical workflows. Using the OpenSlide library, each WSI was subdivided into non-overlapping 512 × 512 tiles, a patch size commonly used in computational pathology to balance local context with computational efficiency [13–15].

To ensure that the model was trained only on informative tissue regions, we applied a two-step filtering strategy to discard background and artifacts. First, a primary intensity filter was applied, where tiles with over 60% of pixels having a grayscale intensity ≥ 220 were discarded as uninformative background. Second, a secondary content filter was used to further exclude tiles containing excessive blank space or scanning artifacts, specifically removing those where bright white or dark black pixels covered ≥ 85% of the area. This preprocessing pipeline ensured that only tiles containing diagnostically relevant tissue structures were retained for model training and evaluation.

### Compression format and settings

To simulate a range of real-world storage scenarios, each tile set was saved in three distinct formats. First, TIFF (Uncompressed) served as the raw baseline format directly extracted from the WSI. Second, PNG (Lossless) was selected as the practical baseline for deep learning workflows. Since PNG employs lossless compression that preserves the exact pixel values of the original WSI—making it mathematically identical to uncompressed TIFF—it ensures zero information loss. Therefore, it serves as the ideal reference standard, offering the same data fidelity as TIFF but with superior storage efficiency and broader compatibility with machine learning libraries. Finally, JPEG2000 (Lossy) was chosen for its tunable compression rates. JPEG2000 is a widely adopted compression standard supported by the DICOM standard for Whole Slide Imaging [16]. Unlike standard JPEG, it utilizes wavelet-based compression, which prevents blocky artifacts and supports efficient region-of-interest (ROI) access for gigapixel images. Furthermore, previous comparative studies have demonstrated that JPEG2000 preserves quantitative cellular features more effectively than standard JPEG formats in automated analysis tasks [17]. Therefore, focusing on this format provides the most clinically relevant insights for WSI storage standardization.

For JPEG2000, we tested ten compression rates: 1, 5, 10, 15, 20, 25, 30, 40, 60, and 80, spanning from near-lossless to highly compressed conditions. The rate parameter specifies the target compression ratio defined by the original bits per pixel (bpp) divided by the compressed bpp. For instance, a rate of 10 targets a file size one-tenth of the original.

### Model architecture and training strategy

#### Deep learning models

To verify the generalizability of our findings across different architectures and complexities, we employed four distinct segmentation models, categorized into two main groups.

First, for the CNN-based U-Net architectures, we trained the widely adopted U-Net [18] using three different backbones to evaluate the robustness of convolutional neural networks (CNNs) with varying depths. MobileNetV3-Large [19] was selected as a representative efficient backbone designed for lightweight applications, allowing us to assess performance under resource-constrained scenarios. Additionally, we utilized ResNet-18 [20] as a standard lightweight CNN backbone and ResNet-50 [20] as a deeper CNN backbone representing a widely used performance benchmark. Second, to incorporate state-of-the-art architectures, we employed the Transformer-based SegFormer (MiT-B1) [21]. This model utilizes a hierarchical Transformer encoder, allowing us to assess whether attention-based mechanisms respond differently to compression artifacts compared to CNNs.

All models were trained with full supervision using expert-annotated binary mask images. To strictly evaluate the robustness of the model against compression artifacts, training was performed exclusively using unmodified, lossless images (PNG format). No compressed data was included in the training set. We used a hybrid loss function combining Dice loss and binary cross-entropy loss to address class imbalance. Training was performed for up to 50 epochs with early stopping (patience = 5) based on validation Dice scores. A batch size of 192 was used, and optimization employed the Adam optimizer (initial learning rate = 1 × 10⁻³) with a StepLR scheduler. All experiments were conducted on an NVIDIA A100 GPU with 80 GB of memory.

#### Cross-validation protocol

To ensure a robust evaluation, we implemented a slide-level 3-fold cross-validation process. The 86 WSIs were randomly divided into three non-overlapping folds consisting of approximately 44 slides for training; 24 slides for validation; and 18 slides for testing.

Each fold yielded approximately 200,000 training tiles, 100,000 validation tiles, and approximately 64,000 test tiles. Slide-level partitioning ensured that no tiles from the same WSIs were shared between the folds, thereby preventing data leakage.

### Evaluation metrics

To comprehensively assess the feasibility of JPEG2000 compression in digital pathology, we evaluated the results from three distinct perspectives: deep learning model performance, image quality, and storage efficiency.

#### Deep learning model performance

To measure the segmentation fidelity of the deep learning model, we utilized the Dice coefficient, which quantifies the pixel-wise overlap between the predicted mask and the ground truth mask. The model output (probability map) was thresholded at 0.5 to generate a binary segmentation mask for each tile. The tile-level Dice score was calculated by comparing this binary mask against the expert-annotated ground truth.

$$Dice = \frac{2\mid P \cap G \mid}{\mid P \mid + \mid G\mid}$$

#### Image quality assessment

We evaluated the visual fidelity of the compressed images using both Full-Reference (FR) and No-Reference (NR) metrics to quantify distortion relative to the original images and perceptual quality, respectively. All image quality metrics were computed on a per-tile basis for the independent test dataset, and the reported values represent the macro-average across all test tiles.

For Full-Reference metrics, since the original uncompressed images were available as ground truth, we employed Peak Signal-to-Noise Ratio (PSNR) and Structural Similarity Index Measure (SSIM). PSNR measures pixel-based reconstruction error, where values ≥ 30 dB are typically considered visually acceptable [4]. SSIM evaluates perceptual changes in luminance, contrast, and structure [22]. Values near 1.0 indicate high fidelity, and previous research using the Just Noticeable Difference (JND) paradigm suggests that compressed images are often visually indistinguishable from the original at high SSIM levels (e.g., > 0.95) [23].

Additionally, to assess perceptual quality without relying on a reference image, we measured the Perception based Image Quality Evaluator (PIQE) as a No-Reference metric. PIQE calculates a quality score based on block-wise distortion estimation; unlike FR metrics, a lower PIQE score indicates better image quality [24]. According to the PIQE quality scale derived from the LIVE Image Quality Assessment Database [25], image quality is categorized as follows: Excellent (0–20), Good (21–35), Fair (36–50), Poor (51–80), and Bad (81–100).

#### Storage efficiency

The total disk space required to store all test tiles under each compression condition was measured. We calculated the percentage reduction in file size compared to the original uncompressed TIFF format to quantify storage efficiency.

## Results

### Tile-level segmentation performance under JPEG2000 compression

To evaluate the effect of compression on segmentation performance, we first established a performance baseline using lossless PNG images. We compared four distinct deep learning architectures: a Transformer-based model (SegFormer) and U-Net models equipped with three different backbones (MobileNetV3-Large, ResNet-18, and ResNet-50). This comparison ensures that the chosen baseline model possesses sufficient capacity to handle lung adenocarcinoma segmentation tasks before assessing compression artifacts.

As summarized in Table 1, the U-Net with a MobileNetV3 backbone achieved the highest average Dice score of 0.827 across the 3-fold cross-validation, outperforming both the larger ResNet-50 backbone (0.779) and the SegFormer (0.816). These results indicate that the lightweight MobileNetV3 architecture captures histological features effectively without the need for heavier computational overhead.

**Table 1:** Baseline segmentation performance (Dice coefficient) of four deep learning models evaluated on lossless PNG images

Fold	SegFormer	U+MobileNetV3	U+ResNet-50	U+ResNet-18
Fold 0	0.8326	0.8468	0.7891	0.7556
Fold 1	0.8042	0.8144	0.7644	0.7410
Fold 2	0.8102	0.8194	0.7843	0.7705
Average	0.816	0.827	0.779	0.756

The Dice scores represent the segmentation performance on the validation set for each fold using uncompressed (lossless) images. The values indicate that the U-Net with MobileNetV3 backbone consistently achieved the highest accuracy across all folds

To evaluate the effect of compression on segmentation performance, quantitative analysis was conducted on the independent test slide set. We calculated the Dice coefficient by comparing the model-predicted segmentation masks against the expert-annotated Ground Truth (GT) masks for each compression scenario.

**Fig. 1:**
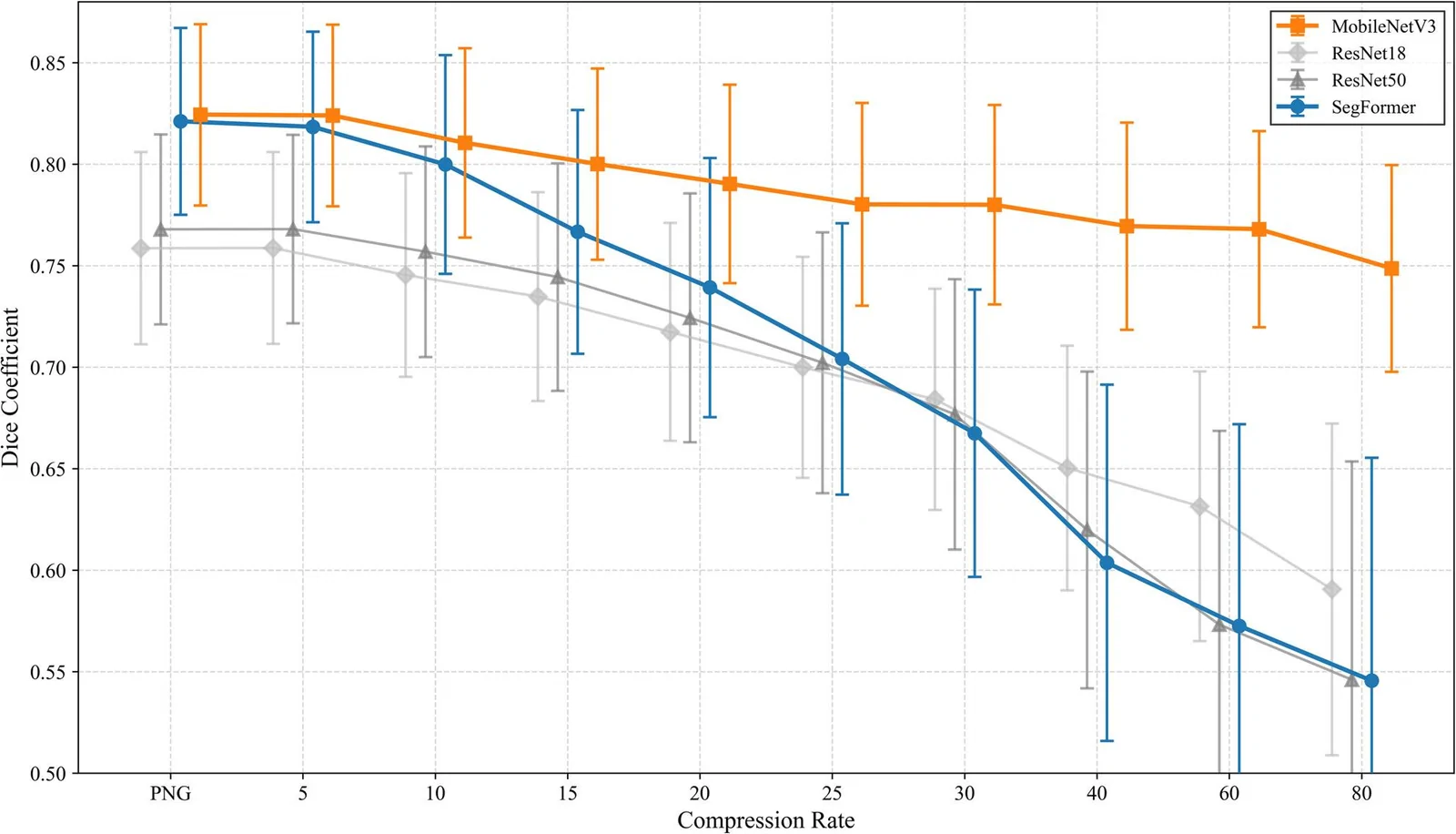
Impact of JPEG2000 compression on segmentation performance across different model architectures. The line plot illustrates the mean Dice coefficients for MobileNetV3 (orange square), ResNet18 (light gray diamond), ResNet50 (dark gray triangle), and SegFormer (blue circle) at varying compression rates. Error bars represent the 95% confidence intervals (CI) derived from 3-fold cross-validation. While the Transformer-based SegFormer shows a steep decline in performance beyond a rate of 10, the MobileNetV3-based U-Net demonstrates superior stability

Figure 1 and Table 2 present the segmentation performance across varying JPEG2000 compression rates. Under lossless conditions (PNG), the U-Net with a MobileNetV3 backbone achieved the highest Dice score of 0.8244. As the compression rate increased, all models exhibited a gradual decline in segmentation accuracy. However, the MobileNetV3-based model demonstrated superior robustness compared to other architectures.

**Table 2:** Quantitative comparison of tile-level segmentation performance (Dice coefficient) across deep learning architectures under varying JPEG2000 compression rates

Image format	SegFormer	U+MobileNetV3	U+ResNet-50	U+ResNet-18
PNG (lossless)	0.8211	0.8244	0.7679	0.7586
JPEG2000 Rate = 1	0.8211	0.8244	0.7679	0.7586
JPEG2000 Rate = 5	0.8184	0.8240	0.7680	0.7587
JPEG2000 Rate = 10	0.7999	0.8105	0.7569	0.7454
JPEG2000 Rate = 15	0.7667	0.8001	0.7444	0.7348
JPEG2000 Rate = 20	0.7393	0.7903	0.7243	0.7174
JPEG2000 Rate = 25	0.7041	0.7802	0.7022	0.7000
JPEG2000 Rate = 30	0.6675	0.7800	0.6768	0.6842
JPEG2000 Rate = 40	0.6037	0.7695	0.6199	0.6504
JPEG2000 Rate = 60	0.5727	0.7680	0.5732	0.6315
JPEG2000 Rate = 80	0.5456	0.7487	0.5461	0.5906

Data are presented as mean Dice coefficients calculated on the independent test set. Models were trained using lossless PNG images and evaluated on JPEG2000-compressed tiles. The segmentation accuracy remained stable up to a compression rate of 10, but a distinct performance decline was observed starting at a rate of 15, particularly in the SegFormer and ResNet-50 models

Up to a compression rate of 10, the MobileNetV3 model recorded a Dice score of 0.8105, showing only a marginal decline of approximately 1.7% from the lossless baseline. In contrast, performance degradation became more pronounced in other models from a rate of 15 onwards. The SegFormer model, in particular, showed high sensitivity to compression artifacts, with its Dice score dropping significantly to 0.7667 at Rate 15 (-6.6%). Notably, at extreme compression rates (Rate 80), while the ResNet-50 and SegFormer models suffered severe performance drops (falling below 0.60), the MobileNetV3 model retained a relatively high Dice score of 0.7487. This indicates that while compression inevitably reduces accuracy, the lightweight MobileNetV3 architecture is significantly more resilient to the loss of high-frequency details.

### Storage savings and cost efficiency

**Fig. 2:**
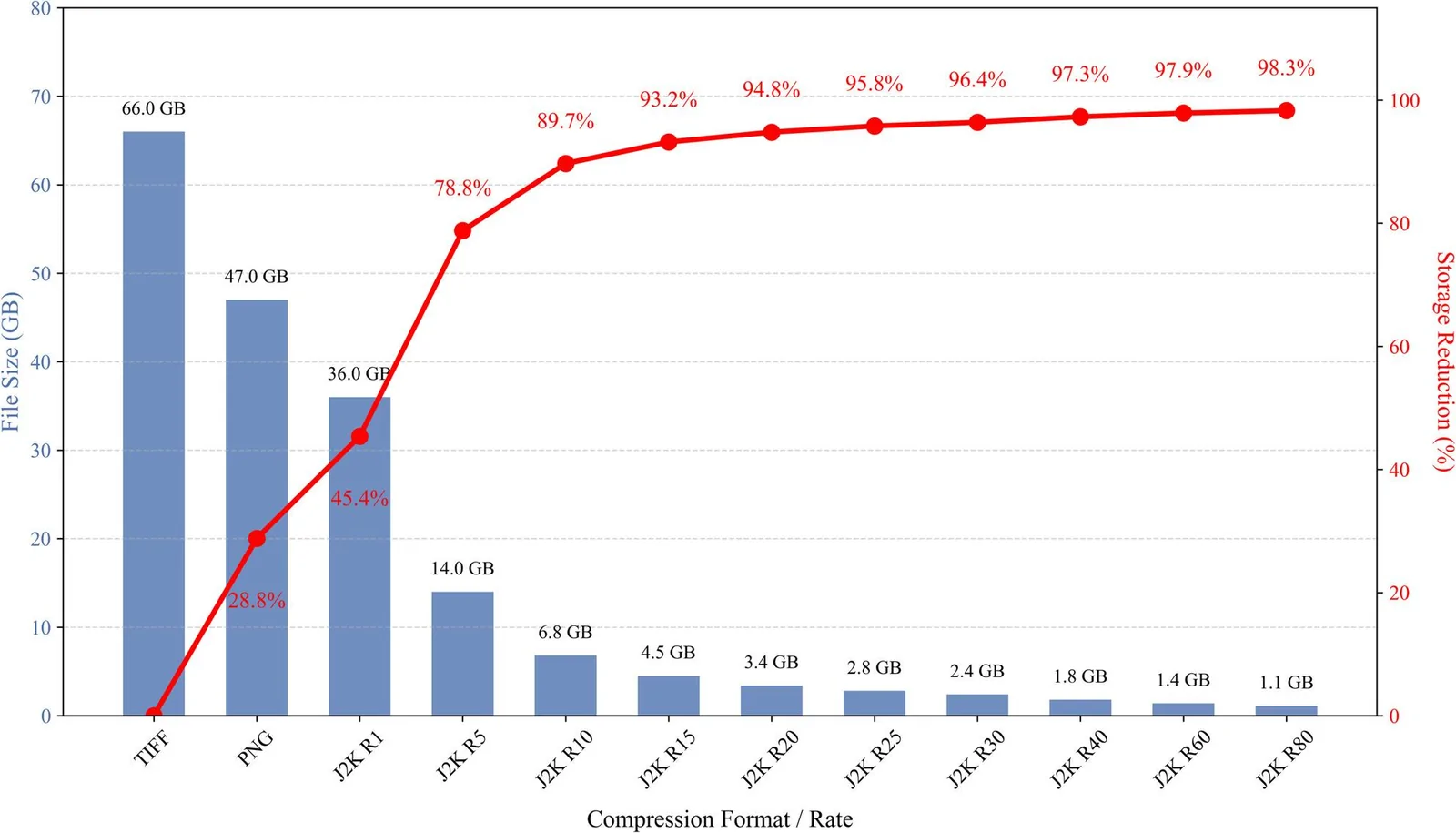
Comparison of storage efficiency and file size reduction across different compression formats. The blue bars (left axis) represent the total file size (GB) required to store tissue-valid tiles extracted from the independent test slides. The red line (right axis) illustrates the cumulative storage reduction percentage (%) relative to the uncompressed TIFF format. Values above the bars and line markers indicate the exact file size and reduction rate, respectively. While the file size decreases exponentially as the compression rate increases, the storage efficiency gain begins to plateau beyond a rate of 10. (TIFF: Tagged Image File Format; PNG: Portable Network Graphics; J2K: JPEG2000)

Figure 2 illustrates the relationship between the JPEG2000 compression rate, storage size, and storage reduction percentage. The storage analysis was performed on tissue-valid tiles extracted from the independent test dataset, excluding non-informative background regions to reflect a practical storage scenario.

The prediction accuracy remained largely stable up to a compression rate of 10, whereas the file size decreased significantly. For example, at a rate of 10, the total storage size for the test set decreased from 66.0 GB (uncompressed TIFF) to 6.8 GB, representing a reduction of approximately 89.7%. The lossless PNG format achieved a reduction of roughly 28.8% (47.0 GB). Notably, JPEG2000 at a rate of 1, which is treated as mathematically lossless, reduced the storage by 45.4% (36.0 GB) while maintaining identical segmentation performance.

At intermediate rates, storage dropped further to 4.5 GB (~ 93.2% reduction) at a rate of 15 and 2.8 GB (~ 95.8% reduction) at a rate of 25. However, these reductions were accompanied by notable accuracy losses (Dice score decline of approximately 3.3% and 10.4%, respectively). At the highest compression rate of 80, the storage usage dropped to 1.1 GB (over 98% reduction), but this came at the cost of substantial performance degradation.

### Image quality degradation analysis

**Fig. 3:**
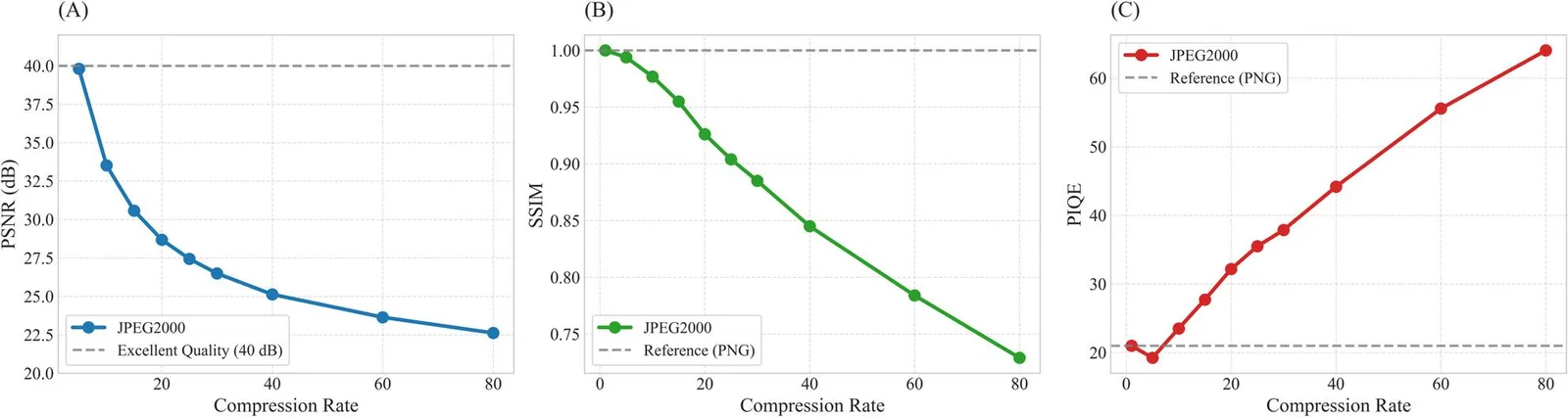
Quantitative impact of JPEG2000 compression on image quality. The graphs display the trends of (**A**) PSNR, (**B**) SSIM, and (**C**) PIQE across varying compression rates. In (**A**), the data point for Rate 1 is omitted as lossless compression results in a mathematically infinite PSNR. The dashed gray line in (**A**) indicates the threshold for excellent image quality (40 dB), whereas the dashed lines in (**B**) and (**C**) represent the baseline values of the uncompressed reference images. Notably in (**C**), the PIQE score at Rate 5 is lower (better) than the reference, suggesting a potential denoising effect

To evaluate the impact of JPEG2000 compression on image fidelity, we analyzed three quantitative metrics—PSNR, SSIM, and PIQE—at each compression rate. As shown in Table 3 and Fig. 3, image quality degraded progressively as the compression rate increased.

**Table 3:** Assessment of image quality metrics (PSNR, SSIM, and PIQE) at varying JPEG2000 compression rates

JPEG2000 (rate = X)	PSNR(dB)	SSIM	PIQE
JPEG2000 (rate = 1)	-	1.0	21.02
JPEG2000 (rate = 5)	39.80	0.994	19.24
JPEG2000 (rate = 10)	33.52	0.977	23.53
JPEG2000 (rate = 15)	30.57	0.955	27.72
JPEG2000 (rate = 20)	28.69	0.926	32.18
JPEG2000 (rate = 25)	27.44	0.904	35.51
JPEG2000 (rate = 30)	26.49	0.885	37.88
JPEG2000 (rate = 40)	25.13	0.845	44.19
JPEG2000 (rate = 60)	23.64	0.784	55.57
JPEG2000 (rate = 80)	22.62	0.729	64.04

The metrics were calculated on tissue-valid tiles from the test dataset. PSNR (Peak Signal-to-Noise Ratio) and SSIM (Structural Similarity Index) are higher-is-better metrics, while PIQE (Perception based Image Quality Evaluator) is a lower-is-better metric. The PIQE score at Rate 5 indicates slightly better perceptual quality than the reference, likely due to noise reduction

At low compression levels (Rate ≤ 10), the images retained high fidelity. For instance, at a compression rate of 5, the PSNR was 39.80 dB and the SSIM was 0.994, indicating negligible distortion. Interestingly, the PIQE score at Rate 5 (19.24) was lower (better) than that of the lossless reference (21.02). Based on the standard PIQE scale, this indicates that mild compression shifted the image quality from the “Good” range (Reference) into the “Excellent” range (Rate 5). This suggests that mild compression may act as a smoothing filter, reducing high-frequency noise that the PIQE algorithm perceives as artifacts.

However, beyond a rate of 10, a clear decline in quality was observed. At Rate 15, the PSNR dropped to 30.57 dB, hovering near the commonly cited acceptability threshold of 30 dB. By Rate 20, the PSNR fell to 28.69 dB and the SSIM decreased to 0.926. These values coincide with the onset of significant segmentation performance degradation observed in our deep learning models.

At higher compression rates (Rate ≥ 40), perceptual quality deteriorated sharply. The PIQE score transitioned into the “Fair” range at Rate 40 (44.19) and further degraded to the “Poor” range at Rate 60 (55.57) and Rate 80 (64.04), reflecting severe blurring and ringing artifacts. This trend aligns with the visual inspection results, where compression artifacts became distinct in tissue areas, particularly disrupting nuclear boundaries and glandular structures.

**Fig. 4:**
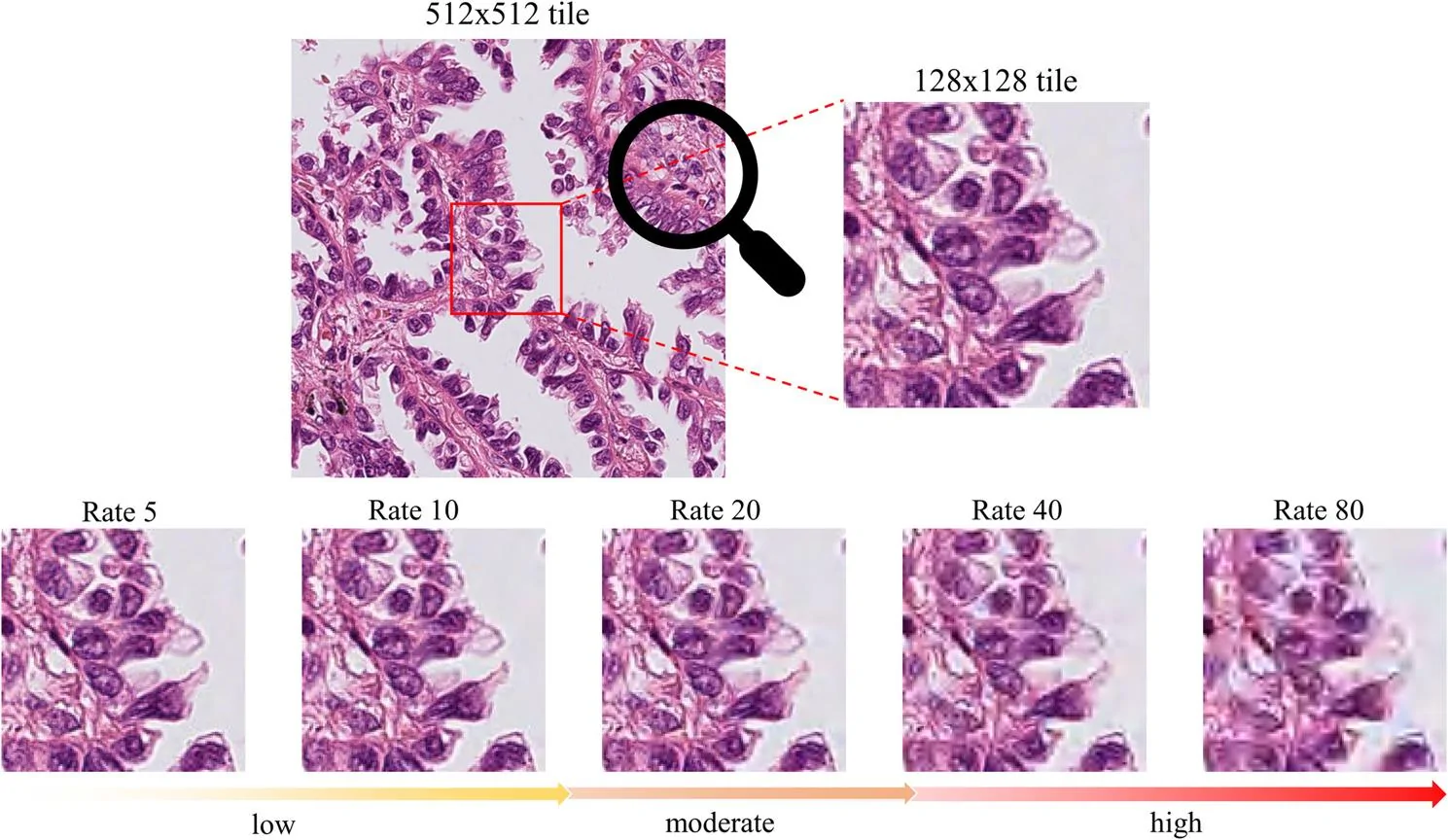
Representative magnified views showing the effect of JPEG2000 compression on fine histological structures. A 512 × 512 tumor tile was extracted from a lung adenocarcinoma WSI, and a 128 × 128 region was magnified for detailed visualization. The bottom row shows the same region compressed at different JPEG2000 rates (5, 10, 20, 40, and 80), with the arrow indicating increasing compression severity from low to high. Fine nuclear and glandular structures are preserved at low compression rates but progressively degrade at higher rates

This trend aligns with the visual inspection results, where compression artifacts became distinct in tissue areas. To clearly demonstrate the effect of compression on fine histological details, Fig. 4 presents a magnified view of a representative tumor tile.

As shown in Fig. 4, fine nuclear boundaries and glandular architectures remain sharp at low compression rates (5 and 10), comparable to the lossless image. However, at moderate compression (Rate 20), slight blurring becomes apparent. At higher compression levels (Rates 40 and 80), fine structures show pronounced artifact patterns and texture loss, which directly challenges the model’s ability to interpret cellular features.

### Analysis of model confidence degradation and compression artifacts

**Fig. 5:**
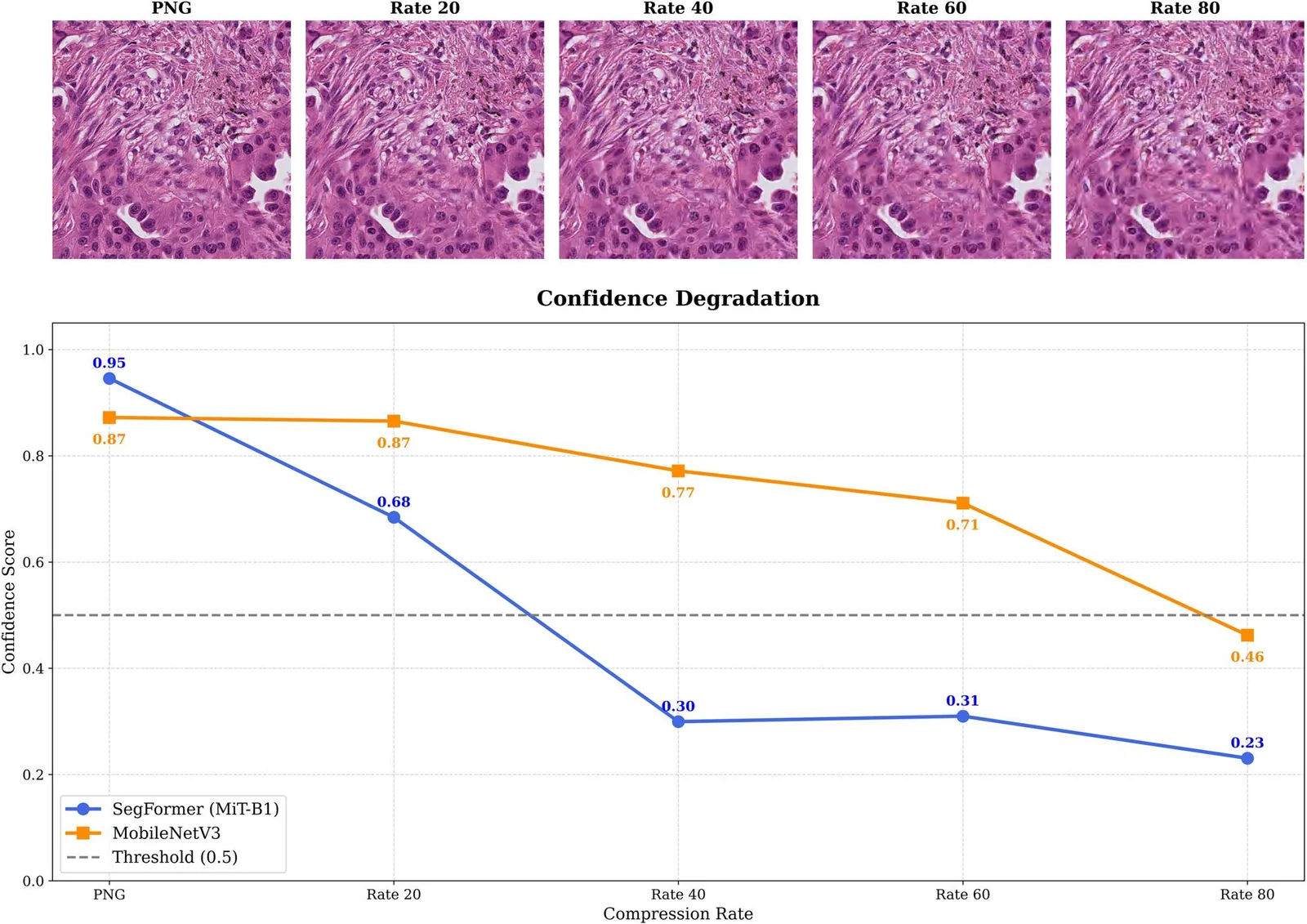
Comparison of visual quality and model confidence degradation between Transformer and CNN architectures. Top: Representative histological image tiles (512 × 512) at increasing compression rates. Visual artifacts such as blurring become distinct at higher rates. Bottom: Line graph tracking the prediction confidence scores of the SegFormer (blue, Transformer-based) and MobileNetV3 U-Net (orange, CNN-based). The dashed line indicates the binary classification threshold (0.5). The Transformer-based model shows a rapid decline in confidence, falling below the threshold at Rate 40, whereas the CNN-based model maintains prediction stability (>0.5) up to Rate 60

Since the Dice coefficient is calculated based on binary masks thresholded at 0.5, it may mask the progressive decline in prediction certainty as long as the probability scores remain above this threshold. Therefore, while the visual artifacts are evident, it is crucial to investigate how they affect the model’s internal decision-making before actual segmentation failure occurs. Figure 5 connects the visual quality degradation with the changes in model prediction confidence.

As shown in the top row of Fig. 5, high-frequency details blur progressively at rates above 20. The bottom graph highlights a distinct difference in behavioral stability between the two architectures. The Transformer-based SegFormer exhibited high sensitivity to these artifacts; its confidence score dropped sharply from 0.95 (PNG) to 0.68 at Rate 20, eventually falling below the classification threshold (0.5) at Rate 40. Conversely, the CNN-based MobileNetV3 model demonstrated greater stability, maintaining confidence scores above 0.7 up to Rate 60.

**Fig. 6:**
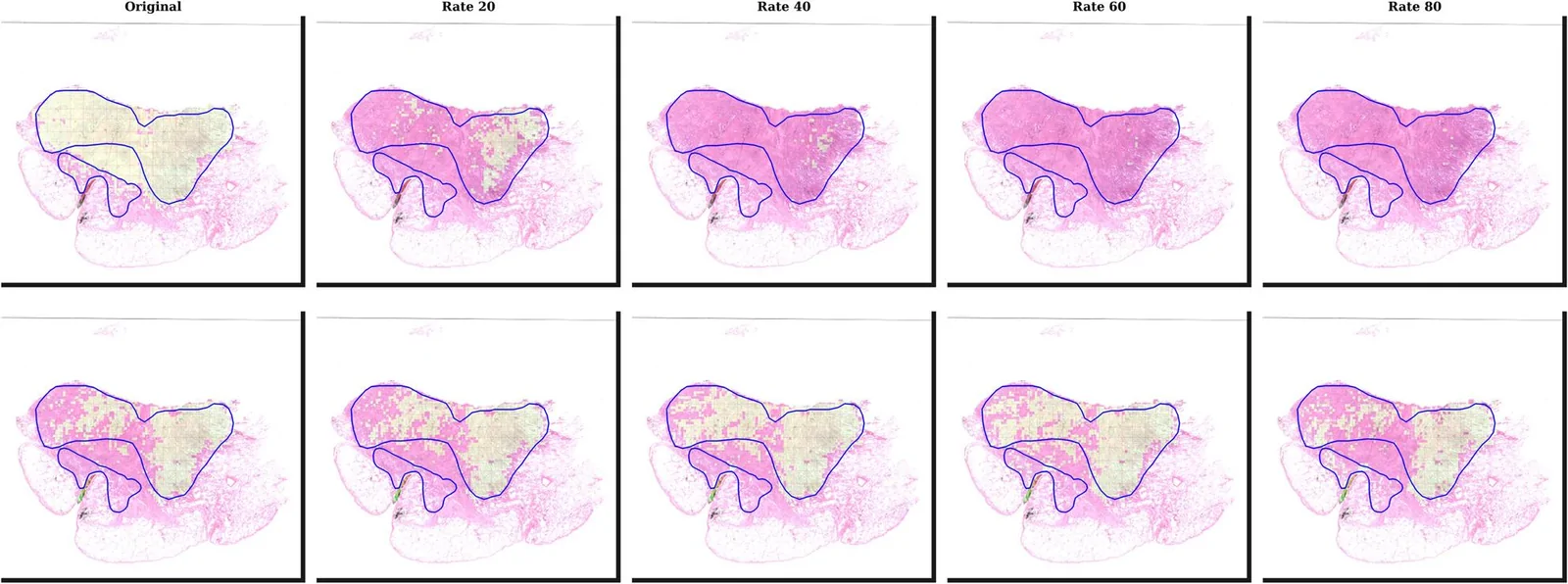
Comparison of segmentation robustness between Transformer and CNN architectures. The segmentation masks (green overlay) are shown against the ground truth tumor boundary (blue contour). Top Row (SegFormer): The model correctly identifies the tumor at the original PNG quality but fails to detect significant tumor regions starting at Rate 40, leading to a near-complete loss of detection at Rate 80. Bottom Row (MobileNetV3 U-Net): The model demonstrates high stability, preserving accurate tumor segmentation even at extreme compression rates (Rate 60 and 80), aligning with its sustained confidence scores

To validate whether this observed drop in confidence translates into actual segmentation failures, we visualized the segmentation masks on whole-slide regions. Figure 6 compares the segmentation results of the Transformer-based SegFormer (top row) and the MobileNetV3-based U-Net (bottom row). Consistent with the confidence analysis in Fig. 5, the SegFormer failed to detect large portions of the tumor area starting at Rate 40. In contrast, the MobileNetV3-based model maintained consistent segmentation boundaries even at high compression rates, demonstrating superior robustness for clinical slide analysis.

## Discussion

This study quantitatively evaluated the relationship between JPEG2000 lossy compression and DL prediction performance, image quality, and storage efficiency using tile-based images extracted from real-world lung adenocarcinoma WSIs. To the best of our knowledge, there is limited research investigating the effects of varying JPEG2000 compression rates on AI performance using real-world clinical lung cancer data.

At level 1 resolution (20× magnification), our results suggest that JPEG2000 compression rates of up to 10 may represent a feasible operating range for tile-level analysis. Within this range, the prediction accuracy remained relatively stable with Dice score differences of less than 2% (approximately 1.7% in the MobileNetV3 model) compared with the lossless PNG. Consistent with Chen et al. [4], PSNR values remained above 33 dB (33.52 dB at Rate 10). Furthermore, PIQE scores maintained an “Excellent” to “Good” quality rating up to Rate 25, which correlates with the stable performance observed in the MobileNetV3 model. However, given that this was a pilot study conducted on a limited dataset from a single institution, these findings should be interpreted as an initial validation rather than a definitive clinical guideline.

Interestingly, at a mild compression rate of 5, the PIQE scores and Dice coefficients were comparable to, or showed a marginal improvement over, the lossless baseline. This phenomenon implies that mild compression did not compromise feature extraction and may have reduced high-frequency acquisition noise due to the low-pass filtering nature of the JPEG2000 Discrete Wavelet Transform (DWT). As noted by Skodras et al. [26], the quantization process in DWT inherently acts as a low-pass filter, effectively suppressing high-frequency acquisition noise while preserving structural information. This suggests that mild compression might serve as a beneficial regularization step, improving model robustness against inherent slide noise without compromising diagnostically relevant features. This observation aligns with established principles in broader bio-image analysis domains, such as microarray image processing, where effective noise reduction and preprocessing are identified as critical prerequisites for achieving accurate segmentation and reliable quantification [27].

A critical finding of this study is the divergent robustness between CNN-based (MobileNetV3) and Transformer-based (SegFormer) architectures. While the MobileNetV3 model maintained relatively stable performance even at high compression rates, the SegFormer exhibited a steep decline starting from compression rates exceeding 10. This disparity likely stems from the differing inductive biases of the two architectures. Dosovitskiy et al. [28] highlighted that CNNs possess strong inductive biases towards locality and translation invariance, enabling them to focus on local textures and shapes even in the presence of noise.

In contrast, Vision Transformers (ViTs) generally lack these inherent biases and rely on global self-attention mechanisms. Recent studies indicate that standard ViTs can be sensitive to image degradations in medical imaging. Bai et al. [29] demonstrated that ViTs effectively function as low-pass filters but may struggle to robustly capture high-frequency components (e.g., edges, fine textures) under corruption. Similarly, Karimijafarbigloo et al. [30] highlighted that standard ViTs struggle with robustness under frequency-domain degradations in medical segmentation tasks. In our study, the loss of high-frequency details (e.g., nuclear edges) due to JPEG2000 compression likely disrupted the patch embeddings essential for the SegFormer’s attention mechanism, leading to the observed confidence drop.

Regarding storage efficiency, lossless formats (PNG) achieved savings of approximately 29%. However, lossy JPEG2000 at moderate rates (5–10) offered superior savings (approx. 80–90%) while maintaining comparable performance within our experimental setting. Since modern WSI formats (e.g., DICOM-WSI, SVS) utilize a multi-resolution pyramid structure composed of tiles, and tissue-containing regions constitute the vast majority of the file size, the compression efficiency observed at the tile level serves as a valid proxy for the reduction in total WSI file size. When the compression rate exceeded 20, the incremental storage reduction became marginal compared to the steep decline in accuracy. This suggests that aggressive compression degrades AI utility disproportionately to the storage gained. These results align with reports that excessive compression introduces artifacts detrimental to computational pathology models.

This study has several limitations. First, although the dataset was expanded, it consists of 86 WSIs collected from a single institution using a single scanner model and staining protocol. Since compression artifacts can vary depending on the digitization nuances and color profiles, broader multicenter validation involving diverse scanners and staining variations is required to ensure generalizability.

Second, the analysis was performed at the tile level using nonoverlapping patches. While this approach effectively quantifies local segmentation fidelity, it does not account for the global spatial context or the slide-level aggregation logic often used in clinical diagnosis. Therefore, the impact of compression on whole-slide diagnostic concordance remains to be fully verified.

Third, our evaluation focused specifically on lung adenocarcinoma. Given that compression tolerance may vary depending on tissue texture and nuclear morphology, these findings may not be directly transferable to other cancer types or non-neoplastic tissues with different histological characteristics.

Fourth, the “trained on lossless only” approach, while valuable for assessing the intrinsic robustness of legacy models, does not address potential performance recovery through domain adaptation or retraining on compressed data.

Finally, the models were trained and evaluated on high-quality consensus annotations refined by experts. In real-world clinical settings, where ground truth data may contain higher label noise, the degradation impact of compression could potentially manifest differently. To address these limitations, future research should prioritize multicenter validation across diverse scanners and cancer types to ensure generalizability. Furthermore, extending the evaluation to whole-slide segmentation and exploring emerging formats like JPEG-XL or WebP will be essential to fully verify the practical applicability of these findings in routine clinical workflows.

## Conclusion

In conclusion, this pilot study investigated the impact of JPEG2000 compression on deep learning-based segmentation using real-world lung adenocarcinoma WSIs. Our results demonstrate that JPEG2000 compression is a highly effective strategy for data management, identifying rates of 5–10 as a viable operating range where storage requirements are reduced by over 90% without compromising segmentation accuracy. Beyond confirming the feasibility of compression, a key contribution of this work is the demonstration of divergent architectural robustness; the CNN-based model exhibited superior intrinsic resilience to compression artifacts compared to the Transformer-based model. These findings provide foundational quantitative data for optimizing data management strategies in digital pathology, suggesting that storage policies can be substantially optimized while being adapted according to the specific AI architectures employed.

## Data Availability

The dataset used in this study is de-identified; however, it was obtained with approval from the Institutional Review Board (IRB) of the affiliated institution and therefore cannot be made publicly available. Access to the dataset may be granted upon reasonable request, subject to approval by both the IRB and the Data Review Board (DRB) in accordance with institutional procedures.The source code for model implementation, image compression, and tile preprocessing is available in a public GitHub repository [31].

## Abbreviations

AI: Artificial intelligence
bpp: Bits per pixel
CI: Confidence interval
CNN: Convolutional neural network
dB: Decibel
DICOM: Digital imaging and communications in medicine
DL: Deep learning
DWT: Discrete wavelet transform
FR: Full-reference
GT: Ground truth
H&E: Hematoxylin and eosin
IHC: Immunohistochemistry
JPEG: Joint photographic experts group
PNG: Portable network graphics
PSNR: Peak signal-to-noise ratio
ROI: Region of interest
SSIM: Structural similarity index measure
PIQE: Perception based Image Quality Evaluator
TIFF: Tagged image file format
U-Net: U-shaped convolutional neural network
ViT: Vision transformer
WSI: Whole-slide image
